# EM modelling of arbitrary shaped anisotropic dielectric objects using an efficient 3D leapfrog scheme on unstructured meshes

**DOI:** 10.1007/s00466-016-1295-x

**Published:** 2016-05-25

**Authors:** A. Gansen, M. El Hachemi, S. Belouettar, O. Hassan, K. Morgan

**Affiliations:** 1 Luxembourg Institute of Science and Technology (LIST), 5, avenue des Hauts–Fourneaux, 4362 Esch/Alzette, Luxembourg; 2 College of Engineering, Swansea University, Bay Campus, Swansea, SA1 8EN UK

**Keywords:** Anisotropic, Co-volume, Finite differences, Unstructured mesh

## Abstract

The standard Yee algorithm is widely used in computational electromagnetics because of its simplicity and divergence free nature. A generalization of the classical Yee scheme to 3D unstructured meshes is adopted, based on the use of a Delaunay primal mesh and its high quality Voronoi dual. This allows the problem of accuracy losses, which are normally associated with the use of the standard Yee scheme and a staircased representation of curved material interfaces, to be circumvented. The 3D dual mesh leapfrog-scheme which is presented has the ability to model both electric and magnetic anisotropic lossy materials. This approach enables the modelling of problems, of current practical interest, involving structured composites and metamaterials.

## Introduction

In anisotropic materials, the electromagnetic material parameters, such as permittivity, permeability and conductivity, may vary in the different crystal directions, so that they must be treated as tensors. It is assumed that already 1000 years ago, before the invention of magnetic compasses, Vikings used crystals, a naturally occuring anisotropic material, in Norse sagas referred to as sunstones to navigate on open water on cloudy days. In accordance to researchers these sunstones could have been calcite crystals where anisotropy leads to the phenomenom of birefrigence (crystalline materials with different indices of refraction with different crystallographic directions). Their sunstone came within 1 % of the true location of the sun [1]. Nowadays anisotropic materials offer many new and interesting perspectives in engineering. A thin anisotropic coating may, for example, significantly change the radar cross section of an aircraft. Composites, anisotropic materials with applications initially limited to stealth bombers, satellites and space shuttles become part of our everyday life. Due to their advantages with respect to mechanical strength and weight compared to metals they are now used in civil aircrafts, trains, automobiles, trucks, sports equipment and so on. Especially in plane and cars electromagnetic compatibility is an issue which can be dealt with using numerical simulations. Other applications are the design of patch antennas where anisotropy can be used as design parameter  [2]. Furthermore anisotropy is the basis of metamaterials, which are new materials with electromagnetic properties which cannot be found in nature, e.g. a material may have a negative index of refraction, which could be employed in the design of invisible cloaking devices [3].

Analytical solutions to wave propagation problems in electromagnetics are mainly restricted to problems involving simple geometrical shapes and diagonal, uniaxial or biaxial, tensors [4, 5]. Numerical techniques are required for the solution of the majority of problems, which involve arbitrary shaped objects. The second order accurate standard Yee algorithm implemented on a pair of staggered orthogonal Cartesian meshes is often the favored computational solution technique because of it’s low operation count and its low storage requirements. Unfortunately in the case of a curved boundary where the physical boundary doesn’t conform to the orthogonal mesh a very fine mesh is required due to errors induced by the stair stepping edges. In earlier work [6], we demonstrated the capability of a generalized Yee algorithm adapted to unstructured meshes to accurately model the radar cross section (RCS) of arbitrarily shaped lossy dielectric objects. For isotropic cases our method shows significant savings with respect to memory and time with respect to the standard FDTD scheme due to the unstructured mesh we employ. Here, we describe the extension of the method to deal with anisotropic materials, such as composites.

There are generally two methods to deal with anisotropic materials. Firstly you use the constitutive equation to replace the displacement field in Maxwells equations by the electric field [7]. Another possibility is to obtain the displacement field and afterwards use it in the constitutive equations. We adopt the latter method which has been proposed by [2]. This approach was originally presented within the context of a total field formulation, but the unstructured mesh extension adopted here employs a scattered field formulation.

## Problem formulation

The integral form of Maxwell’s equations is employed [8]. For a three dimensional lossy dielectric medium, Ampère’s and Faraday’s Laws are expressed, in a scattered field form, as

1 $$\begin{aligned}&\int \limits _{A} \left[ \frac{\partial }{\partial t} +\bar{\bar{\sigma }}{\bar{\bar{\varepsilon }}}^{-1} \right] \mathbf D _{scat} \mathrm {d}\mathbf A = \oint \limits _{\partial A} \mathbf H _{scat}\mathrm {d}\mathbf l \nonumber \\&\quad - \int \limits _{A}({\bar{\bar{\varepsilon }}}-\varepsilon _{0}\bar{\bar{I}}) \frac{\partial \mathbf E _{inc}}{\partial t}\mathrm {d}\mathbf A - \int \limits _{A}\bar{\bar{\sigma }} \mathbf E _{inc}\mathrm {d}\mathbf A \end{aligned}$$

and

2 $$\begin{aligned}&\int \limits _{A} \left[ \frac{\partial }{\partial t} + \bar{\bar{\sigma }}_m \bar{\bar{\mu }}^{-1} \right] \mathbf B _{scat}\mathrm {d}\mathbf A = - \oint \limits _{\partial A}\mathbf E _{scat}\mathrm {d}\mathbf l - \int \limits _{A}(\bar{\bar{\mu }}\nonumber \\&\quad -\mu _{0}\bar{\bar{I}})\frac{\partial \mathbf H _{inc}}{\partial t}\mathrm {d}\mathbf A - \int \limits _{A}\bar{\bar{\sigma _{m}}}\mathbf H _{inc}\mathrm {d}\mathbf A \end{aligned}$$

Here, $\partial A$ denotes a closed curve bounding a surface *A*, $\mathrm {d}\mathbf A$ is an element of surface area, directed normal to the surface, $\mathrm {d}\mathbf l$ is an element of contour length, in the direction of the tangent to the curve, *t* denotes time and $\bar{\bar{I}}$ is the unit matrix. In addition, ${\bar{\bar{\varepsilon }}}$ is the electric permittivity tensor, $\bar{\bar{\mu }}$ is the magnetic permeability tensor, $\bar{\bar{\sigma }}$ and $\bar{\bar{\sigma }}_m$ are the electric and magnetic conductivity tensors respectively and $\varepsilon _0$ and $\mu _0$ denote the electric permittivity and magnetic permeability of free space respectively.The subscripts $(.)_{inc}$ and $(.)_{scat}$ refer to incident and scattered field components, with the total fields regarded as being formed as the sum of the corresponding incident and scattered fields. The vectors $\mathbf D$, $\mathbf E$, $\mathbf B$ and $\mathbf H$ represent the electric flux density, or displacement field, the electric field, the magnetic flux and the magnetic field respectively. The constitutive equations, relating the electric field intensity $\mathbf E$ to the electric flux density $\mathbf D$ and the magnetic field intensity $\mathbf H$ to the magnetic flux density $\mathbf B$, may be expressed as

3 $$\begin{aligned} \mathbf D ={\bar{\bar{\varepsilon }}} \mathbf E \qquad \mathbf B =\bar{\bar{\mu }} \mathbf H \end{aligned}$$

The incident field is assumed to be a monochromatic plane wave, generated by a source located in the far field, which has the form $\mathbf E _{inc}=\mathbf E _{0}\cos (\omega t-\mathbf k \cdot \mathbf r )$, where $\mathbf E _{0}$ is the electric field vector, $\mathbf k$ is the wave vector, $\mathbf r$ is the position vector and $\omega$ is the angular frequency. From the known incident electric field, the incident magnetic field may be determined, using Faraday’s Law, as

4 $$\begin{aligned} \mathbf H _{inc}=\frac{1}{\eta }\hat{\mathbf{k }}\times \mathbf E _{inc} \end{aligned}$$

where $\hat{\mathbf{k }}$ is the unit wave vector and $\eta =\sqrt{\mu _{o}/\varepsilon _{o}}$ is the impedance of free space.

**Fig. 1:**
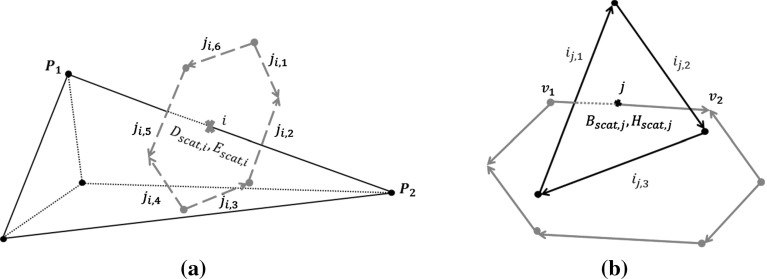
**a** The *i*th Delaunay edge, connecting Delaunay vertices $p_{1}$ and $p_{2}$, and the corresponding Voronoi face, formed by the Voronoi edges $j_{i,1},\ldots ,j_{i,6}$; **b** The *j*th Voronoi edge, connecting Voronoi vertices $v_{1}$ and $v_{2}$, and the corresponding Delaunay face, formed by the Delaunay edges $i_{j,1},\ldots ,i_{i,3}$

## Discrete equations

As the electric and magnetic field vectors are orthogonal, we employ a primal polyhedral mesh, to store the electric field and displacement field projections. The Voronoi dual mesh is used to store the magnetic field and magnetic flux projections. For illustration, we assume here that the primal mesh consists of tetrahedra, generated by a Delaunay method  [9]. By construction, each Voronoi face is a perpendicular bisector of the corresponding Delaunay edge and each Delaunay face is perpendicular to the corresponding Voronoi edge, fulfilling our orthogonality requirements. This means that in an ideal mesh, each Delaunay edge is perpendicular to a surface bounded by a closed loop of Voronoi edges. Similarly, each Voronoi edge is surrounded by a closed loop of Delaunay edges. The Delaunay mesh is assumed to have $N_{e}^{D}$ edges and the Voronoi mesh $N_{e}^{V}$ edges. When the leapfrog scheme is used for time discretization, it will be second order accurate if the unknowns are located at the midpoints of these edges. The unknown at the centre of the *i*th Delaunay edge corresponds to the projection, $(D_{scat,i},E_{scat,i})$, of the scattered electric field onto the direction of the edge, as illustrated in Fig. 1a. The unknown at the centre of the *j*th Voronoi edge corresponds to the projection, $(B_{scat,j},H_{scat,j})$, of the scattered magnetic field onto the direction of the edge, as illustrated in Fig. 1b. In the scattered field formulation, the incident field is a known function, while the scattered field is unknown. At the interface boundaries, the material parameters in the Eqs. (1) and (2) are not constant. In this case, we average the values of ${\bar{\bar{\varepsilon }}}$, $\bar{\bar{\mu }}$, $\bar{\bar{\sigma }}$ and $\bar{\bar{\sigma }}_{m}$ at a dielectric interface, leading to the values ${\bar{\bar{\varepsilon }}}_{av}$, $\bar{\bar{\mu }}_{av}$, $\bar{\bar{\sigma }}_{av}$ and $\bar{\bar{\sigma }}_{m_{av}}$. The method for determining these averaged values is detailed in Sect. 5. Direct discretization of Ampère’s Law and Faraday’s Law then leads to the equations

5 $$\begin{aligned} B_{scat,j}^{n+0.5}= & {} \left\langle \left[ \bar{\bar{I}} + \frac{\triangle t \bar{\bar{\sigma }}_{av_m} \bar{\bar{\mu }}_{av}^{-1}}{2}\right] ^{-1} \left( \left[ \bar{\bar{I}}-\frac{\triangle t \bar{\bar{\sigma }}_{av_m} \bar{\bar{\mu }}_{av}^{-1}}{2}\right] \right. \right. \nonumber \\&\quad \left. \left. \left. \mathbf B _{scat}^{n-0.5} \right| _{j} \right. \right. \nonumber \\&\left. \left. \quad +\,\triangle t \left[ \left( \frac{-1}{A_{j}^D} \sum _{k=1}^{M_{j}^{D}}E_{scat,i_{j,k}}^{n}l_{i_{j,k}}^{D}\right) {\hat{\mathbf{e }}}_{j} -\bar{\bar{\sigma }}_{av_m} \left. \mathbf H _{inc}^{n}\right| _{j}\right. \right. \right. \nonumber \\&\quad \left. \left. \left. - \, \left( \bar{\bar{\mu }}_{av}-\mu _{0}\bar{\bar{I}}\right) \frac{\partial }{\partial t}\left. \mathbf H _{inc}^{n}\right| _{j} \right] \right) , {\hat{\mathbf{e }}}_{j} \right\rangle \end{aligned}$$

and

6 $$\begin{aligned} D_{scat,i}^{n+1}= & {} \left\langle \left[ \bar{\bar{I}} + \frac{\triangle t \bar{\bar{\sigma }}_{av} {\bar{\bar{\varepsilon }}}_{av}^{-1}}{2}\right] ^{-1} \left( \left[ \bar{\bar{I}}-\frac{\triangle t \bar{\bar{\sigma }}_{av} {\bar{\bar{\varepsilon }}}_{av}^{-1}}{2}\right] \left. \mathbf D _{scat}^{n}\right| _{i} \right. \right. \nonumber \\&\left. \left. \quad + \,\triangle t \left[ \left( \frac{1}{A_{i}^{V}} \sum _{k=1}^{M_{i}^{V}}H_{scat,j_{i,k}}^{n+0.5}l_{j_{i,k}}^{V}\right) {\hat{\mathbf{e }}}_{i} - \bar{\bar{\sigma }}_{av} \left. \mathbf E ^{n+0.5}_{inc}\right| _{i}\right. \right. \right. \nonumber \\&\quad \left. \left. \left. - \, \left( {\bar{\bar{\varepsilon }}}_{av}-\varepsilon _{0}\bar{\bar{I}}\right) \frac{\partial }{\partial t}\left. \mathbf E _{inc}^{n+0.5}\right| _{i} \right] \right) , {\hat{\mathbf{e }}}_{i} \right\rangle \end{aligned}$$

where $\triangle t$ denotes the time step, the superscript *n* denotes an evaluation at time level $t=n\triangle t$, $l_{i}^{D}$ represents the length of the *i*th Delaunay edge and $A_{i}^{V}$ corresponds to the area of the Voronoi face spanned by the Voronoi edges surrounding Delaunay edge *i*. Similarly, $l_{j}^{V}$ represents the length of the *j*th Voronoi edge and $A_{j}^{D}$ corresponds to the area of the Delaunay face spanned by the Delaunay edges surrounding Voronoi edge *j*. The numbers $j_{i,k}$, $k=1,\ldots ,M_{i}^{V}$ refer to the $M_{i}^{V}$ edges of the Voronoi face corresponding to the *i*th Delaunay edge, while the numbers $i_{j,k}$, $k=1,\ldots ,M_{j}^{D}$ refer to the $M_{j}^{D}$ edges of the Delaunay face corresponding to the *j*th Voronoi edge. In addition, $\left\langle \mathbf F ,{\hat{\mathbf{e }}_{i}} \right\rangle$ denotes the dot product (projection) of any field vector $\mathbf F$ along the $i^{th}$ edge. We will use both $\left\langle \mathbf F ,{\hat{\mathbf{e }}_{i}} \right\rangle$ and $\mathbf F \cdot {\hat{\mathbf{e }}_{i}}$ to represent the scalar product between a field and a unit edge vector. The projection of the scattered electric field vector onto Delaunay edge *i* is denoted by $E_{scat,i}$, while $\mathbf E _{scat}|_i$ denotes the scattered electric field vector at the location of the *i*th Delaunay edge. Defining the quantities

7 $$\begin{aligned} \bar{\bar{a}}_{\varepsilon +}= & {} \left( \bar{\bar{I}}+\frac{\triangle t \bar{\bar{\sigma }}_{av} {\bar{\bar{\varepsilon }}}_{av}^{-1}}{2}\right) ^{-1}\quad \qquad \bar{\bar{a}}_{\varepsilon -} = \left( \bar{\bar{I}}-\frac{\triangle t \bar{\bar{\sigma }}_{av} {\bar{\bar{\varepsilon }}}_{av}^{-1}}{2}\right) \nonumber \\ \end{aligned}$$

8 $$\begin{aligned} \bar{\bar{a}}_{\mu +}= & {} \left( \bar{\bar{I}}+\frac{\triangle t \bar{\bar{\sigma }}_{av_{m}} \bar{\bar{\mu }}_{av}^{-1}}{2}\right) ^{-1}\qquad \quad \bar{\bar{a}}_{\mu -} = \left( \bar{\bar{I}}-\frac{\triangle t \bar{\bar{\sigma }}_{av_{m}} \bar{\bar{\mu }}_{av}^{-1}}{2}\right) \nonumber \\ \end{aligned}$$

9 $$\begin{aligned} \mathbf Z _B|_j= & {} \triangle t\left[ \left( \frac{-1}{A_{j}^{D}} \sum _{k=1}^{M_{j}^{D}} E_{scat,i_{j,k}}^{n+0.5}l_{i_{j,k}}^{V}\right) {\hat{\mathbf{e }}}_j\right. \nonumber \\&\left. - \left( \bar{\bar{\sigma }}_{{av}_m} \mathbf H ^{n}_{inc}|_j - \left( \bar{\bar{\mu }}_{av}-\mu _{0}\bar{\bar{I}}\right) \frac{\partial }{\partial t} \mathbf H _{inc}^{n}|_j \right) \right] \ \end{aligned}$$

10 $$\begin{aligned} \mathbf Z _D|_i= & {} \triangle t\left[ \left( \frac{1}{A_{i}^{V}} \sum _{k=1}^{M_{i}^{V}} H_{scat,j_{i,k}}^{n+0.5}l_{j_{i,k}}^{V}\right) {\hat{\mathbf{e }}}_i \right. \nonumber \\&\left. \left. \left. - \left( \bar{\bar{\sigma }}_{av} \mathbf E ^{n+0.5}_{inc}\right| _i - \left( {\bar{\bar{\varepsilon }}}_{av}-\varepsilon _{0}\bar{\bar{I}}\right) \frac{\partial }{\partial t} \mathbf E _{inc}^{n+0.5}\right| _i \right) \right] \ \end{aligned}$$

enables us to write Eqs. (5) and (6) as

11 $$\begin{aligned} B_{scat,j}^{n+0.5}= & {} \left\langle \bar{\bar{a}}_{\mu +}^{-1} \left( \bar{\bar{a}}_{\mu -} \mathbf B _{scat}^{n-0.5} |_{j} +\mathbf Z _B|_j \right) , {\hat{\mathbf{e }}}_{j} \right\rangle \end{aligned}$$

12 $$\begin{aligned} D_{scat,i}^{n+1}= & {} \left\langle \bar{\bar{a}}_{\varepsilon +}^{-1} \left( \bar{\bar{a}}_{\varepsilon -} \mathbf D _{scat}^{n} |_{i} + \mathbf Z _D|_i \right) , {\hat{\mathbf{e }}}_{i} \right\rangle \end{aligned}$$

Here, $B_{scat,j}^{n+0.5}$ and $D_{scat,i}^{n+1}$ are projections onto the Delaunay and Voronoi edges respectively, whereas the quantities $\left. \mathbf D _{scat}^{n}\right| _{i}$, $\left. \mathbf E ^{n+0.5}_{inc}\right| _{i}$, and $\left. \mathbf H _{scat}^{n-0.5}\right| _{j}$, $\left. \mathbf H _{inc}^{n}\right| _{j}$ represent field vectors computed at the centre of the *i*th Delaunay edge and the *j*th Voronoi edge respectively. These field values have to be determined from their corresponding stored projections and this calculation, which is not direct, is described in detail in Sect. 6. In contrast to the isotropic case, these equations cannot be updated in one step, as vector-matrix multiplications are involved. These staggered equations are used to advance the solution in a leapfrog manner. The magnetic field is updated over the dual mesh at the half time step, using Eq. (11), and the electric field is updated over the primal mesh at the full time step, using Eq. (12).

The scheme is based upon the projections of the field unknowns at the edge centres. This allows us to use unstructured meshes and to model electromagnetic scattering, even for objects of arbitrary shape. Full details of the mesh generating process that is employed, to ensure Delaunay and Voronoi meshes with the correct properties, may be found elsewhere [8–10]. Here, the additional challenge that is faced, is that we have no direct access to the full field vectors $\mathbf B$, $\mathbf H$ $\mathbf D$ and $\mathbf E$ at a given location. Nevertheless, we are able to get accurate values for the approximated field vectors, that are needed for the matrix-vector multiplication of the updating equations, and the results are then projected to the corresponding Delaunay edge ${\hat{\mathbf{e }}}_{i}$ or Voronoi edge ${\hat{\mathbf{e }}}_{j}$. This updating process is explained in detail in Sect. 8.

## Mesh generation

Structured meshes that are employed for wave propagation problems are generally constructed to have a uniform element size, $\delta$, that is related to the characteristic wavelength, $\lambda$, of the problem. A value of $\delta$ in the range $\lambda /30$ to $\lambda /10$ is typical for practical applications of the Yee scheme on regular cartesian grids [11]. Consider now the problem of generating a body fitted mesh of this form for use with the co-volume algorithm outlined above. The computational domain surrounding a general scattering obstacle is discretised employing a hybrid mesh, which is generated in four stages. In the first stage, an unstructured triangulation of the surface of the scatterer is produced [12] and the triangulation is then placed inside a hexahedral box. The region inside this box is discretised using a regular cartesian mesh of cubes, of a prescribed edge length $\delta$. Cubes within a prescribed distance of the scatterer, or lying internal to the scatterer, are removed in a second stage, to create a staircase shaped surface that completely encloses the scatterer. In the third stage, a point distribution is specified to completely cover the unmeshed region, with those points from the distribution that lie either inside the scatterer or outside the staircase surface being removed. The fourth stage consists of using the points that remain to generate an unstructured tetrahedral mesh in the region between the surface triangulation of the scatterer and the staircase surface. The problem of fitting the hexahedral and tetrahedral meshes is overcome by placing a pyramidal element on each exposed square face, leading naturally to a consistent mesh. The unstructured mesh is optimised to ensure that both the primal and the dual mesh are of the highest possible quality. The approach adopted is to relax the requirement that a dual edge must be a bisector of the corresponding Delaunay edge. At the same time, the corresponding dual mesh vertex is moved to a point which still ensures orthogonality between the two grids and which lies inside the corresponding primal element. Primal elements with a common circumcentre, and hence a corresponding Voronoï edge length of zero, will automatically be merged during the solution process, creating general polyhedral cells.

## Boundary conditions

**Fig. 2:**
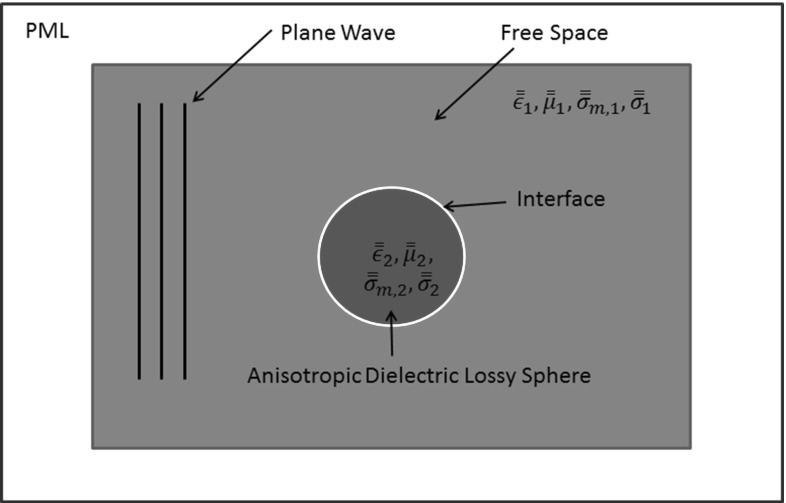
Simulation of scattering by a 3D dielectric sphere in free space

In scattering problems, the incident wave is assumed to be generated by a source located in the far field and the physical solution domain is infinite in extent. The numerical simulation of the scattering problem is undertaken on a finite computational domain. For example, the computational domain employed for the problem of simulating scattering by an anisotropic dielectric sphere, located in free space, is illustrated in Fig. 2. The infinite real physical domain has been truncated and, at the truncated outer computational boundary, the scattered field should only consist of outgoing waves only. The modelling of this requirement is achieved by adding a wave damping perfectly matched layer (PML) [13] to the truncated exterior far-field boundary. In earlier work [14, 15], we have shown that it is convenient to represent the PML region with an assembly of regular hexahedral computational cells.

**Fig. 3:**
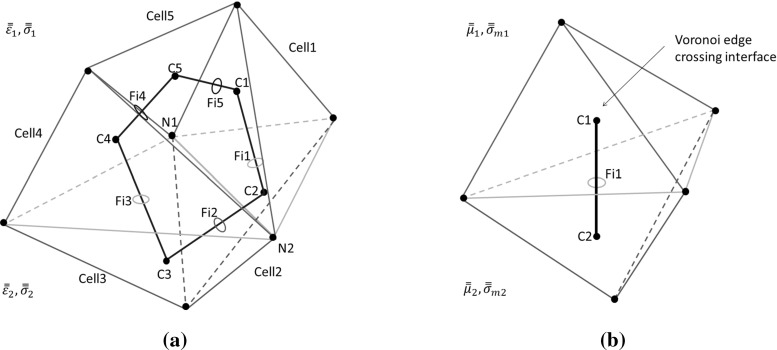
Averaging of material properties at interfaces: **a** Delaunay edges, with the electric area integral crossing the interface, shown in *red*; **b** Voronoi edges, with the magnetic area integral evaluated on the interface, shown in *red*

### PEC boundary conditions

In a scattered field formulation, the condition

13 $$\begin{aligned} \mathbf n \times \mathbf E _{scat} = -\mathbf n \times \mathbf E _{inc} \end{aligned}$$

is applied at the surface of a perfect electric conductor (PEC). Here, $\mathbf n$ is the unit outward normal vector to the PEC surface. Without changing Eqs. (11) and (12), we can strongly impose the electric field unknowns, at the set of edges forming the PEC interface, to satisfy the condition of Eq. (13). Within this leapfrog scheme, we can also model thin resistive or PEC sheets, by assigning the sheet conductivity only to the Delaunay edges forming the interface.

### Material interface boundary conditions

When the boundary is an interface between two different media, the update Eqs. (1) and (2) require integration across the interface. These integrals are evaluated by assigning a weighted average value to the material parameters, based upon the mesh structure.

In a previous publication [6], we obtained material parameters at the interface by a weighted arithmetic mean average and compared the results produced with those obtained by using the arithmetic mean average, the harmonic mean average and the geometric mean average. We demonstrated that the weighted arithmetic mean average resulted in improved accuracy on unstructured meshes. Here, we adopt the same form of averaging, but applied now to every component of the material parameter tensors. In the isotropic case, the scalar material properties, $\varepsilon$ and $\sigma$, associated to the electric field, are stored on the Delaunay edges and the scalars $\mu$ and $\sigma _{m}$ associated to the magnetic field are stored on the Voronoi edges. For an anisotropic material, the scalars $\varepsilon$, $\sigma$, $\mu$ and $\sigma _{m}$ become second order tensors ${\bar{\bar{\varepsilon }}}$, $\bar{\bar{\mu }}$, $\bar{\bar{\sigma }}$ and $\bar{\bar{\sigma }}_{m}$ and, for each component of these tensors, we employ the averaging used in the isotropic case. In Fig. 3a, b, the colour code employed in the diagram of Fig. 2 is adopted, with blue indicating Delaunay edges in medium (1), green indicating Delaunay edges in medium (2) and red the Delaunay edges forming the interface. The Voronoi edges, surrounding a given Delaunay edge at the interface, are indicated in black. In Eqs. (2) and (1), the quantities ${\bar{\bar{\varepsilon }}}$, $\bar{\bar{\mu }}$, $\bar{\bar{\sigma }}$ and $\bar{\bar{\sigma }}_{m}$ lie inside the integrals. In the discretized Eqs. (12) and (11), each component is averaged over the closed surface loop and the averaged values can be taken outside the integrals. For these Delaunay and Voronoi edges, each component (*q*, *l*) of the material property tensors is evaluated, using weighted average formulae, as

14 $$\begin{aligned} \left. {\varepsilon _{av}}_{q,l}\right| _{Del,i}= & {} \frac{\sum \nolimits _{k=1}^{2M^V_i}w_{k} \left. {\varepsilon _{q,l}}\right| _{Cell,k} }{\sum \nolimits _{k=1}^{2M^V_i}w_{k}} \nonumber \\ \left. {\sigma _{av}}_{q,l}\right| _{Del,i}= & {} \frac{\sum \nolimits _{k=1}^{2M_i^V}w_{k} \left. {\sigma _{q,l}}\right| _{Cell,k} }{\sum \nolimits _{k=1}^{2M_i^V}w_{k}} \end{aligned}$$

Here *q* and *l* can take the values 1, 2 or 3, corresponding to the *x*, *y* or *z* directions respectively, while $M_i^V$ refers to the number of Voronoi edges surrounding a given Delaunay Edge *i*. As there are two sub-volumes associated to each Voronoi edge, we have to sum over $2M_i^V$ Voronoi edges. These account for the contribution of the material parameter assigned to each of the cells surrounding Delaunay edge $(.)_{Del,i}$, weighted by a coefficient $w_{k}$ that corresponds to the volume spanned by the two endpoints of the Delaunay edge, the intersection point of the Voronoi edge with the Delaunay face and the position of the circumcentre of the cell. For example, to obtain $\left. {\varepsilon _{av}}_{q,l}\right| _{Del,i}$, in Fig. 3a, $w_{1}$ would be the volume spanned by the points *N*1, *N*2, *C*1 and *Fi*1, while $\left. {\varepsilon _{q,l}}\right| _{Cell,1}$ would correspond to the permittivity component of the element *Cell*1. The points labelled *N* belong to the Delaunay mesh, the points labelled *C* are the circumcentres of the corresponding cells and *Fi* refers to the intersection point of the Voronoi edge with a face spanned by Delaunay edges.

**Fig. 4:**
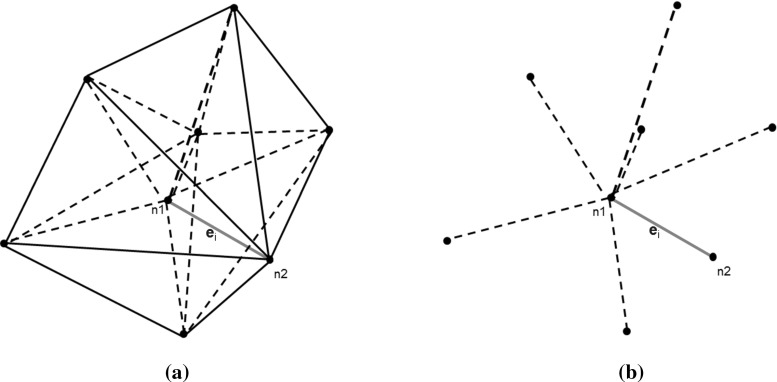
Interpolation of the field vector at node $n_1$: **a** Tetrahedra in the Delaunay mesh containing the point $n_1$; **b** edges in the 3 Delaunay mesh containing the point $n_1$

Each component of the tensors of magnetic permeability $\bar{\bar{\mu }}$ and magnetic conductivity $\bar{\bar{\sigma }}_{m}$, which are linked to the Voronoi edges, is obtained, by averaging, as

15 $$\begin{aligned} \quad \left. {\mu _{av}}_{q,l}\right| _{Vor,j}= & {} \frac{ g_{1} \left. {\mu _{q,l}}\right| _{Cell1} + g_{2} \left. {\mu _{q,l}}\right| _{Cell2} }{g_{1}+g_{2}} \nonumber \\ \left. {{\sigma _{m}}_{av}}_{q,l}\right| _{Vor,j}= & {} \frac{ g_{1} \left. {{\sigma _{m}}_{q,l}}\right| _{Cell1} + g_{2} \left. {{\sigma _{m}}_{q,l}}\right| _{Cell2} }{g_{1}+g_{2}} \end{aligned}$$

The lengths of the Voronoi edges, *g*1 and *g*2, inside *cell*1 and *cell*2 are the distances between the intersection point *Fi*1 of the Voronoi edge $(.)_{Vor,j}$ with the Delaunay face and the circumcentre of the cell. For example, in Fig. 3b, the distance between the points *C*1 and *Fi*1 is $g_{1}$.

## Obtaining approximated field vectors from edge projections

In Sect. 3 it was noted that the main difficulty with Eqs. (11) and (12) are the matrix-vector multiplications. This is because we only store incomplete field vectors, due to our projection based nature of the updating scheme. The challenge is now to obtain the corresponding field vectors $\mathbf D _{scat}$ and $\mathbf B _{scat}$ and associate them with Delaunay edge *i* and Voronoi edge *j* respectively. Unfortunately, we cannot get exact full field vector components from field to edge projections. However, we can approximate the full field components at any location in the mesh. To achieve this, we assume that, in ${\mathbb {R}}^3$, with a set of three orthogonal vectors $\mathbf v _1,\mathbf v _2,\mathbf v _3$, a general vector $\mathbf x$ can be reconstructed as

16 $$\begin{aligned} \mathbf x =\sum \limits _{i=1}^{3} \mathbf P _{v_i} \end{aligned}$$

in terms of the projections

17 $$\begin{aligned} \mathbf P _{v_i}=\frac{<\mathbf x ,\mathbf v _i>\mathbf v _i}{||\mathbf v _i ||^2}\qquad \text{ for }\qquad i=1,2,3 \end{aligned}$$

As the mesh is assumed to be unstructured, we cannot use this method directly to obtain an exact field vector. The trivial solution would be to consider all the edges connected to one node, as depicted in Fig. 4b, and solve a system of equations. This will give us a field vector at node $n_1$. As one edge is always formed by two points, we can do the same for point $n_2$. Finally, we average these field vectors and project to the corresponding edge. For example, to obtain the displacement field vector $\mathbf D _{scat}$ on the edge *i*, denoted by the red line in Fig. 4a, we have to construct the set of equations

18 $$\begin{aligned} \bar{\bar{\mathbf{P }}}\, \mathbf D _{scat}=\left( \mathbf D _{scat} \cdot {\hat{\mathbf{e }}}_i\right) {\hat{\mathbf{e }}}_i \end{aligned}$$

where

19 $$\begin{aligned} \bar{\bar{\mathbf{P }}}= \left[ \begin{array}{l@{\quad }l@{\quad }l} e_{i_x} e_{i_x} &{} e_{i_x} e_{i_y} &{} e_{i_x} e_{i_z} \\ e_{i_y} e_{i_x} &{} e_{i_y} e_{i_y} &{} e_{i_y} e_{i_z} \\ e_{i_z} e_{i_x} &{} e_{i_z} e_{i_y} &{} e_{i_z} e_{i_z} \end{array} \right] \end{aligned}$$

and ${\hat{\mathbf{e }}}_i$ is the normalized Delaunay edge vector corresponding to Delaunay edge *i*. The matrix ${\bar{\bar{\mathbf{P }}}}$, based upon the *x*, *y*, *z* components of the vector ${{\hat{\mathbf{e }}}}_i$ and the projections $\mathbf D _{scat} \cdot {{\hat{\mathbf{e }}}}_i$ are known.

**Fig. 5:**
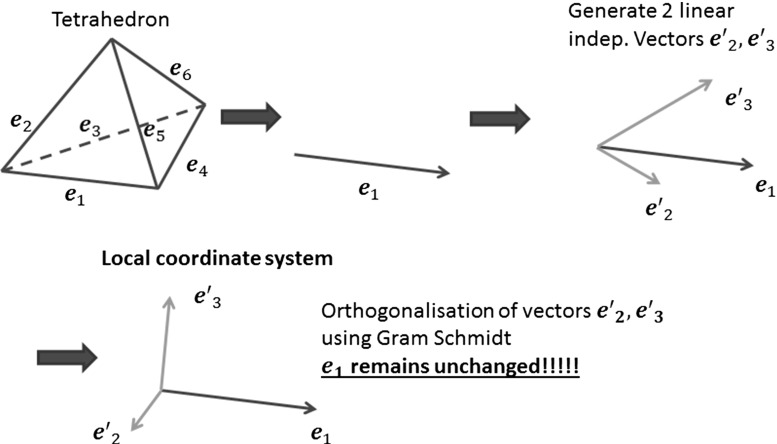
Generating the orthonormal local coordinate system

The displacement field vector at a node belonging to Delaunay edge *i* is approximated by considering the sum of the system in Eq. (18) for each Delaunay edge connected to that node, as depicted in Fig. 4b. In this case, we solve the system

20 $$\begin{aligned} {\bar{\bar{\mathbf{P }}}}' \mathbf D _{scat}=\sum _{q=1}^{N} \left( \mathbf D _{scat,q} \cdot {{\hat{\mathbf{e }}}}_{q}\right) {\hat{\mathbf{e }}}_{q} \end{aligned}$$

with

21 $$\begin{aligned} {\bar{\bar{\mathbf{P }}}}_{l,m}' = \sum _{q=1}^{N} \sum _{l=1}^3 \sum _{m=1}^3 e_{q_l}e_{q_m} \end{aligned}$$

Here, $\mathbf D _{scat}$ is the unknown vector, $\bar{\bar{\mathbf{P }}}'$ and $\mathbf D _{scat,q} \cdot {\hat{\mathbf{e }}}_{q}$ are known, *N* is the number of Delaunay edges connected to the node and $e_{i_1}=e_{i_x}$, $e_{i_2}=e_{i_y}$ and $e_{i_3}=e_{i_z}$. We solve this system of equations locally, node by node, until we have an approximated field vector at all nodes of the Delaunay mesh. Finally, we have to link the computed field vector to the corresponding edges. A Delaunay edge is assumed to connect the two nodes $n_1$ and $n_2$ and the displacement field vector, associated to the Delaunay edge *i*, is obtained by averaging the electric field vectors at nodes $n_1$ and $n_2$, i.e. $\mathbf D _{scat,i}=\left( \mathbf D _{scat,n_1}+\mathbf D _{scat,n_2}\right)$/2. The same procedure is applied for approximating the magnetic flux vectors $\mathbf B _{scat}$, but using now the Voronoi edges. However, unlike the Cartesian Yee scheme, where we have one update equation for each component of the field vectors, the approximations of the full vector fields obtained using this method are not good enough for time iterating the field components. It is found that error accumulation causes the algorithm to become unstable. In the following sections, we suggest how this difficulty may be circumvented.

## Local orthogonal unit vectors

When using the integral formulation of Maxwell’s equations, it is not the displacement field vector $\mathbf D _{scat}$ that is updated, but rather its projection, $D_{scat,i}=\mathbf D _{scat}\cdot \mathbf e _i$, onto a Delaunay edge $\mathbf e _i$. In the case of an isotropic material, the electric permittivity and the magnetic permeability are scalars, so that updating the fields only involves scalar multiplication between the field projections and the scalar material properties. For an anisotropic material, the integrals in Ampère’s and Faraday’s laws contain matrix-vector multiplications between material tensors $({\bar{\bar{\varepsilon }}}, \bar{\bar{\mu }}, \bar{\bar{\sigma }}, \bar{\bar{\sigma }}_{m})$ and the fields $(\mathbf D _{scat},\mathbf B _{scat})$. To deal with these matrix-vector multiplications, we first create two linearly independent vectors for each Delaunay and Voronoi edge. Using the stabilized Gram-Schmidt orthonormalization procedure, we generate three orthonormal vectors, as illustrated in Fig. 5. The first vector $\mathbf e _{1}$, to which the two linearly independent vectors $(\mathbf e ^{\prime }_{2}, (\mathbf e ^{\prime }_{3})$ are added, remains unchanged during the whole process. Each set of three orthogonal vectors represents one local coordinate system, leading to as many local systems as Delaunay and Voronoi edges. Due to the discretisation, we can reconstruct approximated field vector components for each local coordinate system, using the field projections of the surrounding Delaunay or Voronoi edges, as explained in Sect. 6. The field vectors, obtained in this way, are projected onto the two orthogonal vectors forming the local frame. The first vector of each subset, which remains unchanged during the orthonormalization procedure, can be immediately updated using the projection equation employed in the isotropic case. For this projection, no error is induced by the field averaging.

### Coordinate transformation

The material tensors are expressed in the global reference frame formed by the orthonormal vectors ${\hat{\mathbf{x }}}$, ${\hat{\mathbf{y }}}$ and ${\hat{\mathbf{z }}}$. Three orthonormalized vectors ${\hat{\mathbf{x }}}',\ {\hat{\mathbf{y }}}',\ {\hat{\mathbf{z }}}'$ form the basis of each local coordinate system. The $( \hat{.} )$ always refers to unit vectors and $(.')$ to vectors or vector components of the local coordinate system. The Jacobian matrix ${\bar{\bar{J}}}$, defined by

22 $$\begin{aligned} {\bar{\bar{J}}}= \left[ \begin{array}{l@{\quad }l@{\quad }l} \frac{\partial x'}{\partial x}&{} \frac{\partial x'}{\partial y} &{} \frac{\partial x'}{\partial z} \\ \frac{\partial y'}{\partial x} &{}\frac{\partial y'}{\partial y} &{}\frac{\partial y'}{\partial z} \\ \frac{\partial z'}{\partial x} &{}\frac{\partial z'}{\partial y} &{}\frac{\partial z'}{\partial z} \end{array} \right] \end{aligned}$$

can be used to transform a vector, or a matrix, from the global to a local frame. Here $(x',y',z')$ refers to the coordinate in the local coordinate system and (*x*, *y*, *z*) to the global coordinate system. Each component of the Jacobian can be interpreted as an amplification factor, describing how one coordinate in a given reference frame stretches, shrinks or rotates with respect to another coordinate in another reference frame. In our case, the Jacobian is pure a rotation matrix ${\bar{\bar{J}}}_R$ which can be directly calculated as

23 $$\begin{aligned} {\bar{\bar{J}}}_R= \left[ \begin{array}{l@{\quad }l@{\quad }l} {\hat{\mathbf{x }}}'\cdot {\hat{\mathbf{x }}} &{}\quad {\hat{\mathbf{x }}}'\cdot {\hat{\mathbf{y }}} &{} \quad {\hat{\mathbf{x }}}'\cdot {\hat{\mathbf{z }}} \\ {\hat{\mathbf{y }}}'\cdot {\hat{\mathbf{x }}} &{}\quad {\hat{\mathbf{y }}}'\cdot {\hat{\mathbf{y }}} &{} \quad {\hat{\mathbf{y }}}'\cdot {\hat{\mathbf{z }}} \\ {\hat{\mathbf{z }}}'\cdot {\hat{\mathbf{x }}} &{}\quad {\hat{\mathbf{z }}}'\cdot {\hat{\mathbf{y }}} &{} \quad {\hat{\mathbf{z }}}'\cdot {\hat{\mathbf{z }}} \end{array} \right] \end{aligned}$$

Maxwell’s equations are form invariant [16, 17], which means that, in the local coordinate system, they may be expressed as

24 $$\begin{aligned} \int \limits _{A'} {\bar{\bar{\varepsilon }}}' \frac{\partial }{\partial t} \mathbf E '\mathrm {d}A' = \oint \limits _{\partial A}' \mathbf H '\mathrm {d}l', \quad \int \limits _{A'} \bar{\bar{\mu }}' \frac{\partial }{\partial t} \mathbf H ' \mathrm {d}A' = \oint \limits _{\partial A'} \mathbf E '\mathrm {d}l' \nonumber \\ \end{aligned}$$

The electric and magnetic fields, in local and global coordinates, are related

25 $$\begin{aligned} \mathbf E '(\mathbf r ')=({\bar{\bar{J}}}_R^{\ T})^{-1} \mathbf E (\mathbf r ), \qquad \mathbf H '(\mathbf r ')=({\bar{\bar{J}}}_R^{\ T})^{-1} \mathbf H (\mathbf r ) \end{aligned}$$

and a material parameter tensor, ${\bar{\bar{M}}}$ say, is transformed into the local coordinate system using the operator transformation

26 $$\begin{aligned} {\bar{\bar{M}}}'(\mathbf r ')=\frac{{\bar{\bar{J}}}_R {\bar{\bar{M}}} {\bar{\bar{J}}}_R^{\ T}}{\text{ det }({\bar{\bar{J}}}_R)} \end{aligned}$$

Due to the form invariance, it follows that we can absorb the coordinate transformation completely into the material properties. Note that the determinant of the rotation matrix is unity, i.e. $\text{ det }({\bar{\bar{J}}}_R)=1$.

## Time updating scheme

As mentioned previously, Eqs. (11) and (12) cannot be updated simultaneously, as the scattered field vectors $\mathbf D _{scat}$ and $\mathbf B _{scat}$ are not immediately available. In this section, the updating process will be explained in detail. For simplicity, we will restrict consideration to updating the electric field projection $E^n_{scat}$, as the magnetic field $H^n_{scat}$ will be similarly updated. We first apply the coordinate transformation to the terms $\bar{\bar{a}}_{\varepsilon +}, \bar{\bar{a}}_{\varepsilon -}$ defined in  (7) and ${\bar{\bar{\varepsilon }}}_{av}'^{-1}$ from Eq. (12), so that

27 $$\begin{aligned} {\bar{\bar{a}}}'_{\varepsilon +}= & {} {\bar{\bar{J}}}_R \left( \bar{\bar{I}}+\frac{\triangle t \bar{\bar{\sigma }}_{av} {\bar{\bar{\varepsilon }}}_{av}^{-1}}{2}\right) ^{-1} {\bar{\bar{J}}}^{\ T}_R,\end{aligned}$$

28 $$\begin{aligned} {\bar{\bar{a}}}'_{\varepsilon -}= & {} {\bar{\bar{J}}}_R \left( \bar{\bar{I}}-\frac{\triangle t \bar{\bar{\sigma }}_{av} {\bar{\bar{\varepsilon }}}_{av}^{-1}}{2}\right) \ {\bar{\bar{J}}}^{\ T}_R,\end{aligned}$$

29 $$\begin{aligned} {\bar{\bar{\varepsilon }}}_{av}'^{-1}= & {} {\bar{\bar{J}}}_R \ {\bar{\bar{\varepsilon }}}_{av}^{-1} \ {\bar{\bar{J}}}^{\ T}_R \end{aligned}$$

These are stored at the corresponding Delaunay edges before entering the time loop. Within the time iteration loop, we first calculate and store the right hand side of the Eq. (10) for each Delaunay edge ${\hat{\mathbf{e }}}_i$. This is readily accomplished, as the magnetic components in the circulation term $\sum _{k=1}^{M_{i}^{V}}H_{scat,j_{i,k}}^{n+0.5}l_{j_{i,k}}^{V}$ are available, from the previous iteration, and the full vector $\mathbf{E }^{n+0.5}_{inc,i}$ is also a known function. Before updating the projection $D_{scat}^n$ to $D_{scat}^{n+1}$, Eq. (20) is employed, to obtain the vectors $\mathbf D _{scat}^n$ and $\mathbf Z _D$ as defined above . These vectors are projected in each of the three orthogonal directions of every local frame, e.g. ${\hat{\mathbf{e }}}'_1\equiv {\hat{\mathbf{e }}}_1,\ {\hat{\mathbf{e }}}'_2,\ {\hat{\mathbf{e }}}'_3$ from Fig. 5, and the matrix vector multiplication is performed. Equation (12) in the local frame takes the vector form

30 $$\begin{aligned} \mathbf{D '}_{scat}^{n+1}={\bar{\bar{a}}}'_{\varepsilon } + \left[ \bar{\bar{a}}'_{\varepsilon -}\mathbf{D '}_{scat}^n + \mathbf Z '_D\right] \end{aligned}$$

We can use the constitutive equation to obtain the electric field projection. In practice, we only have to consider the first line of ${\bar{\bar{\varepsilon }}}'^{-1}_{av}$, due to the fact that our data storage is based upon field projections along the edges and not field vectors. As a result, the value

31 $$\begin{aligned} E_{scat,i}^{n+1}= & {} {\bar{\bar{\varepsilon }}}_{av,11}^{-1}D_{scat,e_1}^{'n +1}+{\bar{\bar{\varepsilon }}}_{av,12}'^{-1}D_{scat,e'_2}^{'n +1}\nonumber \\&\quad +\,{\bar{\bar{\varepsilon }}}_{av,13}'^{-1}D_{scat,e'_3}^{'n+1} \end{aligned}$$

may be obtained. These values are used for updating the sum up curl term of Eq. (9) and then the magnetic field is updated in a similar manner.

**Fig. 6:**
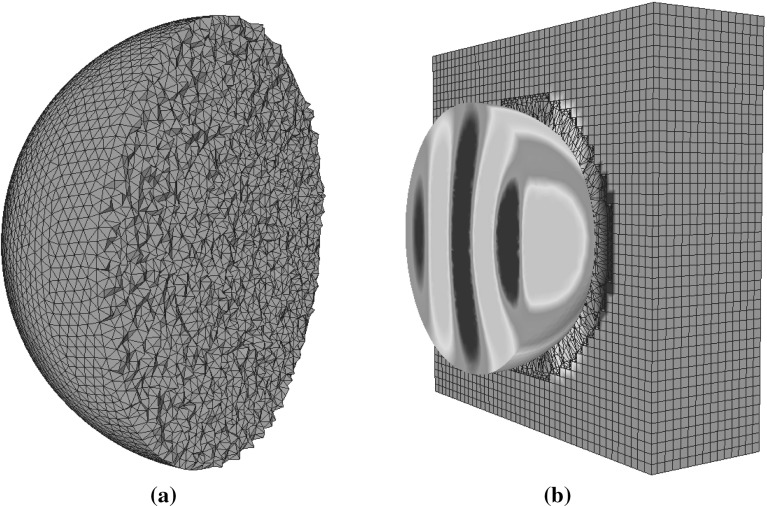
Scattering by a dielectric anisotropic sphere: **a** cut through the mesh used to represent the sphere; **b** contours of $E_y$ on a cut through the computational domain

## Validation

A series of examples, involving scattering of an incident plane wave by an anisotropic sphere, is included here to demonstrate the numerical performance of the algorithm that has been described. The algorithm is validated by comparing the results produced with those obtained from the open source program Discrete Dipole Scattering (DDSCAT) [18], which is an implementation of the frequency domain discrete dipole approximation [19]. The incident wave has free space wavelength $\lambda _0=1m$ and it propagates in the *x* direction. In each case, the electrical length of the sphere is $2\lambda _0$. The mesh employed is illustrated in Fig. 6 and has an average edge length of $\lambda _0/20$. For each example, the PML region is located at a minimum distance of $\lambda _0$ from the scatterer and the PML is discretised using 10 layers of hexahedra. The minimum distance between the inner boundary of the PML and the surface of the scatterer is represented by 8 cells. The complete mesh consists of 876, 116 cells, 1, 673, 527 Delaunay edges and 2, 076, 019 Voronoi edges. The distribution of the radar cross section of the cross- and co-polarized scattered waves is computed as

32 $$\begin{aligned} \chi = \lim _{r\rightarrow \infty } 4\pi r^{2} \frac{\left| {\breve{E}}_{scat,\theta }\right| ^{2}+\left| {\breve{E}}_{scat,\phi }\right| ^{2}}{\left| {\breve{E}}_{inc ,\theta }\right| ^{2}+\left| {\breve{E}}_{inc ,\phi }\right| ^{2}} \end{aligned}$$

where the phasor amplitudes ${\breve{E}}$ and ${\breve{H}}$, expressed in spherical coordinate system $(r, \theta , \phi )$, are calculated by taking the Fourier transform of the time domain solution. The quantity displayed in each case is the radar cross section

33 $$\begin{aligned} RCS(\theta ,\phi )=10\log _{10}(\chi ) \end{aligned}$$

Due to the spherical symmetry of these examples, we only display the RCS values in the range $0^{\circ }$ to $180^{\circ }$. Further details of the RCS computation and the nature of the mesh can be found elsewhere [6].

It is worth noting that our unstructured mesh scheme only needs 8 points per wavelength to obtain accurate results in free space [20]. However, inside a dielectric, the wavelength $\lambda _{Diel}$ is less than the wavelength in free space $\lambda _0$. For the anisotropic case, it is estimated that a mesh spacing of $\lambda _0/20$ mesh should be sufficient. As we include a test case with full anisotropic tensors for both electric and magnetic properties, we decided to use the same mesh for all the test cases.

### Magnetically uniaxial non-lossy anisotropic sphere

**Fig. 7:**
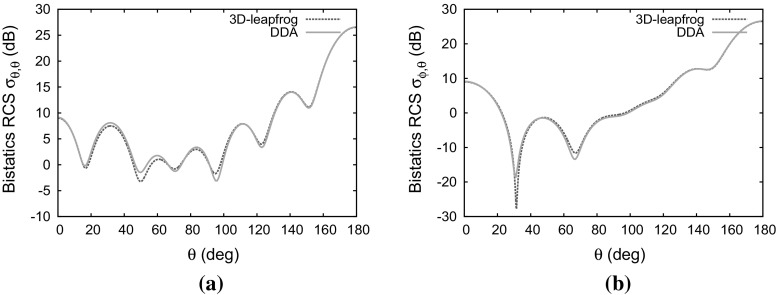
Scattering of a plane wave by a dielectric sphere of electrical length $2\lambda$ with anisotropic permeability: **a** co-polarized RCS distribution; **b** cross-polarized RCS distribution

The first test case involves an uniaxial permeability tensor and is devised to check the updating of the magnetic field projections. For the sphere, the material parameters

34 $$\begin{aligned} {\bar{\bar{\varepsilon }}}_r= & {} \left[ \begin{array}{c@{\quad }c@{\quad }c} 1 &{} 0 &{} 0 \\ 0 &{} 1 &{} 0 \\ 0 &{} 0 &{} 1 \end{array} \right] \qquad \bar{\bar{\sigma }}= \left[ \begin{array}{c@{\quad }c@{\quad }c} 0 &{} 0 &{} 0 \\ 0 &{} 0 &{} 0 \\ 0 &{} 0 &{} 0 \end{array} \right] \nonumber \\ \bar{\bar{\mu }}_r= & {} \left[ \begin{array}{c@{\quad }c@{\quad }c} 1.5 &{} 0 &{} 0 \\ 0 &{} 1.5 &{} 0 \\ 0 &{} 0 &{} 2.0 \end{array} \right] \qquad \bar{\bar{\sigma }}_m= \left[ \begin{array}{c@{\quad }c@{\quad }c} 0 &{} 0 &{} 0 \\ 0 &{} 0 &{} 0 \\ 0 &{} 0 &{} 0 \end{array} \right] \end{aligned}$$

are used. In this case the only term in Eq. (5) that involves matrix multiplication is $(\bar{\bar{\mu }}_{av}-\mu _{0}\bar{\bar{I}}\ )\frac{\partial }{\partial t}\left. \mathbf H _{inc}^{n}\right| _{j}$. The matrices $\bar{\bar{a}}'_{\mu +}$ and $\bar{\bar{a}}'_{\mu -}$ reduce to the unit matrix and the update of of magnetic field from the constitutive Eq. (31) only requires multiplication by the coefficient $(\bar{\bar{\mu }}^{'-1}_{av})_{11}$. Figure 7 shows the computed distributions of both the cross-polarised ($\sigma _{\theta \phi }$) and the co-polarised ($\sigma _{\theta \theta }$) RCS compared with the distributions obtained from using the discrete dipole approximation (DDA). It can be seen that the RCS distributions are in excellent agreement, apart from the differences in the troughs, which are typical of comparisons between time-domain and frequency-domain approximations.

**Fig. 8:**
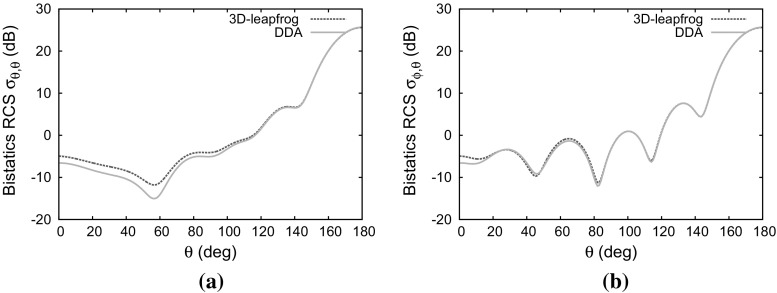
Scattering of a plane wave by a dielectric sphere of electrical length $2\lambda$ with anisotropic permittivity: **a** co-polarized RCS distribution ; **b** cross-polarized RCS distribution

### Electrically uniaxial non-lossy anisotropic sphere

This test case involves a uniaxial permittivity tensor, to check on the updating of the electric field projections. For this case, the material parameter values

35 $$\begin{aligned} {\bar{\bar{\varepsilon }}}= & {} \left[ \begin{array}{l@{\quad }l@{\quad }l} 1.5 &{} 0 &{} 0 \\ 0 &{} 1.5 &{} 0 \\ 0 &{} 0 &{} 2.0 \end{array} \right] \qquad \bar{\bar{\sigma }}= \left[ \begin{array}{l@{\quad }l@{\quad }l} 0 &{} 0 &{} 0 \\ 0 &{} 0 &{} 0 \\ 0 &{} 0 &{} 0 \end{array} \right] \nonumber \\ \bar{\bar{\mu }}= & {} \left[ \begin{array}{l@{\quad }l@{\quad }l} 1 &{} 0 &{} 0 \\ 0 &{} 1 &{} 0 \\ 0 &{} 0 &{} 1 \end{array} \right] \qquad \bar{\bar{\sigma }}_m= \left[ \begin{array}{l@{\quad }l@{\quad }l} 0 &{} 0 &{} 0 \\ 0 &{} 0 &{} 0 \\ 0 &{} 0 &{} 0 \end{array} \right] \end{aligned}$$

are used for the sphere.

In this case the only term that require matrix multiplication in Eq. (6) is $({\bar{\bar{\varepsilon }}}_{av}-\varepsilon _{0}\bar{\bar{I}}\ )\frac{\partial }{\partial t}\left. \mathbf E _{inc}^{n+0.5}\right| _{j}$, as the other matrices $\bar{\bar{a}}'_{\varepsilon +}$ and $\bar{\bar{a}}'_{\varepsilon -}$ reduce to the unit matrix. The update of the electric field from the constitutive Eq. (31) only involves multiplication by the coefficient $({\bar{\bar{\varepsilon }}}^{'-1}_{av})_{11}$. Figure 8 shows good agreement between the computed RCS distributions and those produced with the DDA method. There seems to be a big difference between our solution and the solution from DDA for the co-polarized RCS distribution in Fig. 8a but this is mainly due to the logarithmic scaling we are using highlighting even small differences between the results.

### Electrically lossy anisotropic sphere

**Fig. 9:**
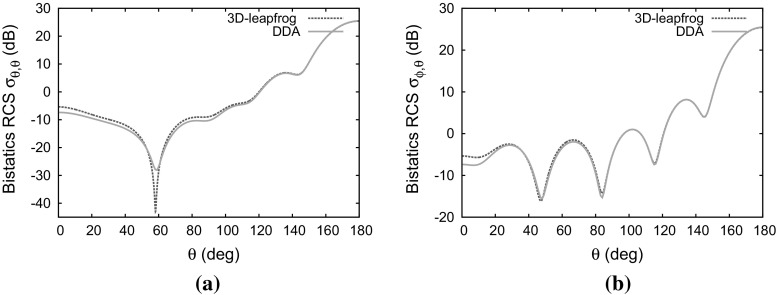
Scattering of a plane wave by a dielectric sphere of electrical length $2\lambda$ with anisotropic permittivity and electrical conductivity: **a** co-polarized RCS distribution; **b** cross-polarized RCS distribution

The next example involves an anisotropic permittivity tensor, together with electric conductivity. The material parameters

36 $$\begin{aligned} {\bar{\bar{\varepsilon }}}= & {} \left[ \begin{array}{l@{\quad }l@{\quad }l} 1.3 &{} 0 &{} 0 \\ 0 &{} 1.6 &{} 0 \\ 0 &{} 0 &{} 2.0 \end{array} \right] \qquad \bar{\bar{\sigma }}= \left[ \begin{array}{l@{\quad }l@{\quad }l} 0.3 &{} 0 &{} 0 \\ 0 &{} 0.5 &{} 0 \\ 0 &{} 0 &{} 0.7 \end{array} \right] \nonumber \\ \bar{\bar{\mu }}= & {} \left[ \begin{array}{l@{\quad }l@{\quad }l} 1 &{} 0 &{} 0 \\ 0 &{} 1 &{} 0 \\ 0 &{} 0 &{} 1 \end{array} \right] \qquad \bar{\bar{\sigma }}_m= \left[ \begin{array}{l@{\quad }l@{\quad }l} 0 &{} 0 &{} 0 \\ 0 &{} 0 &{} 0 \\ 0 &{} 0 &{} 0 \end{array} \right] \end{aligned}$$

are employed. In this case, the matrix multiplications are required in Eq. (6) and the time updating scheme described in Sect. 8 was used. The RCS distributions computed with the 3D-leapfrog and with the DDA scheme are compared in Fig. 9. Steady state conditions were attained in the time domain approach after ten cycles of the incident wave.

**Fig. 10:**
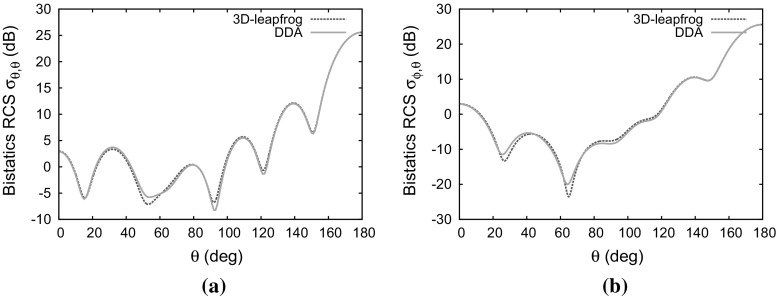
Scattering of a plane wave by a dielectric sphere of electrical length $2\lambda$ with anisotropic permeability and magnetic conductivity: **a** co-polarized RCS distribution; **b** cross-polarized RCS distribution

### Magnetically lossy anisotropic sphere

For the next example, we consider an anisotropic permeability tensor, together with magnetic conductivity. The sphere, in this case, is characterised by the material parameters

37 $$\begin{aligned} {\bar{\bar{\varepsilon }}}= & {} \left[ \begin{array}{l@{\quad }l@{\quad }l} 1 &{} 0 &{} 0 \\ 0 &{} 1 &{} 0 \\ 0 &{} 0 &{} 1 \end{array} \right] \qquad \bar{\bar{\sigma }}= \left[ \begin{array}{l@{\quad }l@{\quad }l} 0 &{} 0 &{} 0 \\ 0 &{} 0 &{} 0 \\ 0 &{} 0 &{} 0 \end{array} \right] \nonumber \\ \bar{\bar{\mu }}= & {} \left[ \begin{array}{l@{\quad }l@{\quad }l} 1.3 &{} 0 &{} 0 \\ 0 &{} 1.6 &{} 0 \\ 0 &{} 0 &{} 2 \end{array} \right] \qquad \bar{\bar{\sigma }}_m= \left[ \begin{array}{l@{\quad }l@{\quad }l} 0.3 &{} 0 &{} 0 \\ 0 &{} 0.5 &{} 0 \\ 0 &{} 0 &{} 0.7 \end{array} \right] \end{aligned}$$

In this example, matrix multiplication is required in Eq. (5) and the scheme described in Sect. 8 was used for the magnetic field update. The time domain solver reached steady state after ten cycles of the incident wave. The comparison between the RCS distributions computed with the current time domain approach and frequency domain method is given in Fig. 10.

**Fig. 11:**
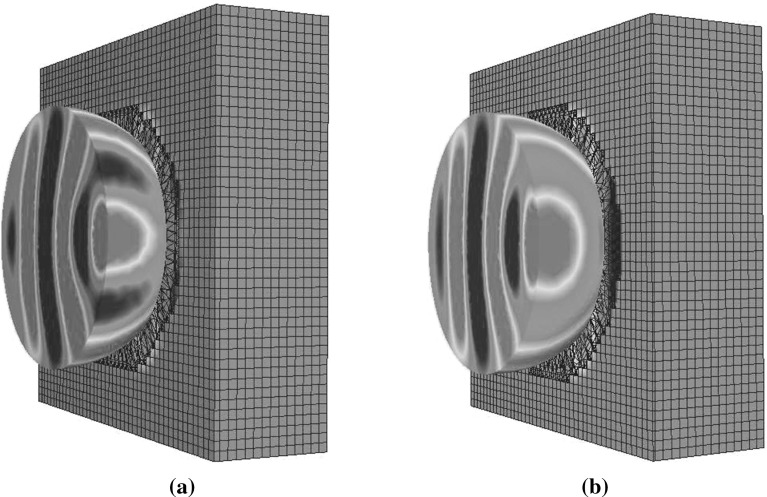
Scattering of a plane wave by a fully anisotropic dielectric sphere of electrical length $2\lambda$: **a** contours of $H_z$ shown on a cross-section through the mesh; **b** contours of $E_y$ shown on a cross section through the mesh

### Computational cost of the anisotropic model

The next example includes the use of full anisotropic tensors for both electric and magnetic properties shown in Fig. 11. The sphere is characterised by the parameters

38 $$\begin{aligned} {\bar{\bar{\varepsilon }}}= & {} \left[ \begin{array}{l@{\quad }l@{\quad }l} 1.3 &{} 0 &{} 0 \\ 0 &{} 1.6 &{} 0 \\ 0 &{} 0 &{} 2.0 \end{array} \right] \qquad \bar{\bar{\sigma }}= \left[ \begin{array}{l@{\quad }l@{\quad }l} 0.3 &{} 0 &{} 0 \\ 0 &{} 0.5 &{} 0 \\ 0 &{} 0 &{} 0.7 \end{array} \right] \nonumber \\ \bar{\bar{\mu }}= & {} \left[ \begin{array}{l@{\quad }l@{\quad }l} 1.2 &{} 0 &{} 0 \\ 0 &{} 1.4 &{} 0 \\ 0 &{} 0 &{} 1.8 \end{array} \right] \qquad \bar{\bar{\sigma }}_m= \left[ \begin{array}{l@{\quad }l@{\quad }l} 0.4 &{} 0 &{} 0 \\ 0 &{} 0.3 &{} 0 \\ 0 &{} 0 &{} 0.5 \end{array} \right] \end{aligned}$$

Comparison with DDA results is not possible in this case, as the DDA code only allows for the modelling of non-magnetic materials. To investigate the time penalty that results from the use of the anisotropic model, two simulations were performed on the same mesh. In the first example, the sphere was taken to be an isotropic lossy dielectric material, with $\mu > 1$, $\varepsilon >1$, $\sigma >0$, $\sigma _m>0$. In the second example, the sphere was modelled as an anisotropic lossy dielectric material, so that $\bar{\bar{\mu }} \ne \mu _r \bar{\bar{I}}$ , ${\bar{\bar{\varepsilon }}}\ne \varepsilon _r \bar{\bar{I}}$ , $\bar{\bar{\sigma }}> [0]_{3\times 3}$ , $\bar{\bar{\sigma _m}}> [0]_{3\times 3}$. It was found that the computational cost for the example involving the anisotropic sphere was ten times the cost of the solution for the isotropic sphere. This extra cost mainly arises from equation (20), which implies a requirement to solve a system of three equations for each Voronoi and Delaunay node. Considering these additional costs, is this scheme competitive when compared to the standard FDTD method? In our first paper [6], we showed that the accuracy of this scheme, with a $\lambda /15$ unstructured mesh, compares favourable with that of the standard FDTD method, with a $\lambda /90, \lambda /120$ structured mesh, for objects of curved shape. This means that we get comparable results when using a mesh that is 6 to 8 times coarser. A standard FTDT Yee’s cell has 12 edges and 6 faces where the electric and magnetic field components are stored respectively. This corresponds to 18 degrees of freedom. Discretising one Yee’s cell requires 6 tetrahedra, in the worst case scenario, leading to 19 edges and 14 faces. As we store the electric field projections at the Delaunay edges and the magnetic field projections at Voronoi edges intersecting with the faces, we then have 33 degrees of freedom in total. Although the number of degrees of freedom have nearly doubled, this is more than compensated by the fact that we use a mesh that is, at least, 6 times coarser. Furthermore, because we are working in three dimensions, this should also be taken into account. This implies that, for the same volume, an unstructured mesh with 33 degrees of freedom produces the same accuracy as a structured mesh with $6^3\times 6 = 1296$ degrees of freedom.

The extra operations, needed for averaging and reconstructing the vectors described in Sect. 6, imply a cost penalty. For each node, we require 18 averaging operations for each field, in addition to $3 \times 3$ operations of vector reconstruction for each edge. For the 6 tetrahedra lying inside one cube, the number of operations will be $2 \times 8 \times 18 + 9 \times (19+14)=585$ for all degrees of freedom, taking into account the 19 electric field vectors and 14 magnetic field vectors. For one cell of a Cartesian mesh, 8 operations are required for averaging the two offset components of a field vector, leading to 48 extra operations on one cell node or 144 per Yee’s cell. However, in our case dealing with curved boundaries, to achieve the same level of accuracy, the standard scheme will actually cost $6^3 \times 48=10368$ operations per same computational volume whereas we only have 585 operations.

### Transmission of a narrow band pulse 

**Fig. 12:**
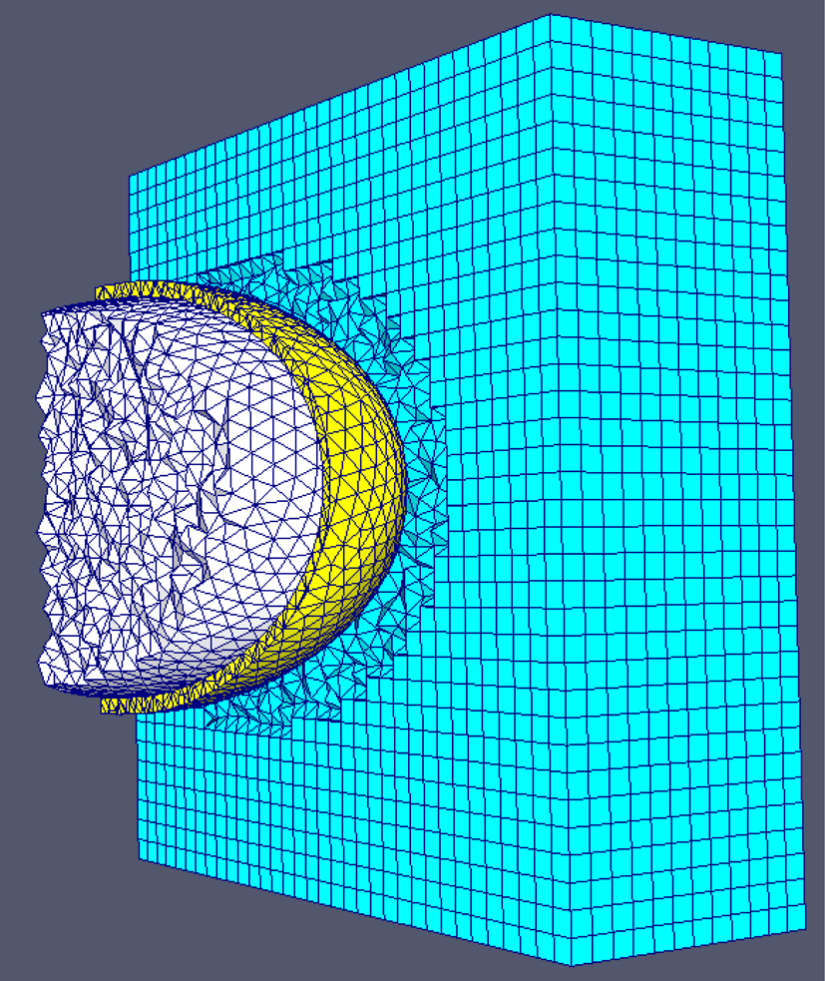
Radome (*yellow*) in free space (*white*, *blue*)

As final example, we evaluate the transmission efficiency of a pulse through a radome made of anisotropic dielectric containing conducting fibres. Radomes of this type are typically used to conceal aircraft communications or radar systems. The radome consists of half an ellipsoid, with a lateral radius of 0.5m, a length of 1m and a thickness of 0.05 m. Figures 12 and 14 display a radome mesh with 20 points per wavelength. This mesh is only used for visualization, as it is too coarse for a 1GHz pulse. For the actual simulation, a finer mesh with 40 points per wavelength is used. In [6], we showed that the number of element layers employed to represent a given thickness of material has no impact on the results, provided that we meet the minimum number of points per wavelength requirement.

**Fig. 13:**
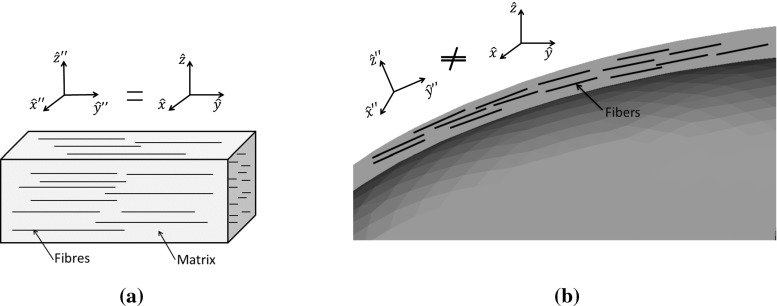
Composite material: **a** material frame **b** Distance between cell centres and material frame located at surface

The incident plane wave used to illuminate the Radome is a narrow band pulse

39 $$\begin{aligned} E_{y}(r,t)=e^{-\frac{(t-k\cdot r/\omega )^{2}}{2\tau ^{2}}} \sin (\omega t-\mathbf k \cdot \mathbf r ) \end{aligned}$$

where $\tau$ denotes the pulse width, $\mathbf k$ is the the wave vector and $\mathbf r$ is the general position vector. Composites are more complicated to model than standard anisotropic materials, due to the inner structure, e.g orientation of the fibres. In Fig. 13a we illustrate a cut through a composite slab with the fibres oriented in the ${\hat{\mathbf{y }}}^{{\prime }{\prime }}$ direction. Due to the specific orientation of the slab, the material frame $({\hat{\mathbf{x }}}'',{\hat{\mathbf{y }}}'',{\hat{\mathbf{z }}}'')$ and the global frame $({\hat{\mathbf{x }}},{\hat{\mathbf{y }}},{\hat{\mathbf{z }}})$ are identical. In the case of the curved shell of a radome, the situation is more complicated, as the orientation of the fibres changes in space. In this case, for each location on the shell, we have a material frame which, in general, differs from the global frame as illustrated in Fig. 13b.

**Fig. 14:**
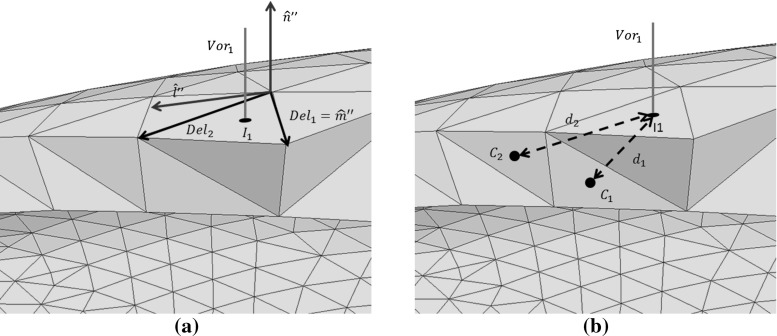
Radome of composite material: **a** Construction of orthonormal material frame. **b** Distance between cell centres and material frame located at surface

In the previous sections, we have only used the coordinate transformation to pass from a global frame $({\hat{\mathbf{x }}},{\hat{\mathbf{y }}},{\hat{\mathbf{z }}})$ to a local frame linked to each Voronoi or Delaunay edge $({\hat{\mathbf{x }}}',{\hat{\mathbf{y }}}',{\hat{\mathbf{z }}}')$. For a composite, we first have to make a coordinate transformation from the material frame, linked to the orientation of the fibres, $({\hat{\mathbf{x }}}'',{\hat{\mathbf{y }}}'',{\hat{\mathbf{z }}}'')$ to the global frame $({\hat{\mathbf{x }}},{\hat{\mathbf{y }}},{\hat{\mathbf{z }}})$. Then we pass from the global frame to a local frame linked to each Voronoi or Delaunay edge, according to $({\hat{\mathbf{x }}}',{\hat{\mathbf{y }}}',{\hat{\mathbf{z }}}')$. $({\hat{\mathbf{x }}}'',{\hat{\mathbf{y }}}'',{\hat{\mathbf{z }}}'') \rightarrow {\bar{\bar{J}}}_{R1} \rightarrow ({\hat{\mathbf{x }}},{\hat{\mathbf{y }}},{\hat{\mathbf{z }}})\rightarrow {\bar{\bar{J}}}_{R2} \rightarrow ({\hat{\mathbf{x }}}',{\hat{\mathbf{y }}}',{\hat{\mathbf{z }}}')$. Here, ${\bar{\bar{J}}}_{Ri}, \; i=1,\,2$, are the two transformation matrices, where ${\bar{\bar{J}}}_{R2}$ is identical to ${\bar{\bar{J}}}_R$ from Eq. (23). First, we have to create an orthonormal material frame. As in the preceding sections, an orthonormal system simplifies the coordinate transformation because the transformation matrix becomes a simple rotation matrix. The procedure here is illustrated in Fig. 14a. Initially, we only consider Voronoi edges at the dielectric interface. Each Voronoi edge (Vor1,blue) crossing the face of a tetrahedron is surrounded by three Delaunay edges. We select two of these edges, ($Del_1$,$Del_2$). By construction, they are parallel to the surface. Using the cross product, we create a vector $\mathbf x ''=Del_1\times Del_2$ perpendicular to the dielectric surface. Finally, we take the cross product to obtain the vector $\mathbf y ''=\mathbf z ''\times Del_1$ and assign $\mathbf z ''=Del_1$. After normalizing, we have one orthonormal material frame $({\hat{\mathbf{x }}}'',{\hat{\mathbf{y }}}'',{\hat{\mathbf{z }}}'')$ for each face of the dielectric interface. The next step consists in linking the material frame to the cells inside the composite. To achieve this, we compare the distance between the intersection points of a Voronoi edge with the interface and the circumcentre of all cells $C_1$ inside the dielectric. After finding the smallest distance, we link the local material frame $({\hat{\mathbf{x }}}',{\hat{\mathbf{y }}}',{\hat{\mathbf{z }}}')$ from the surface to the corresponding cell. This process is illustrated in Fig. 14b.

40 $$\begin{aligned} {\bar{\bar{\mathbf{J }}}}_{R1}= & {} \left[ \begin{array}{l@{\quad }l@{\quad }l} \mathbf x ''\cdot \mathbf x &{} \mathbf x ''\cdot \mathbf y &{} \mathbf x ''\cdot \mathbf z \\ \mathbf y ''\cdot \mathbf x &{} \mathbf y ''\cdot \mathbf y &{} \mathbf y ''\cdot \mathbf z \\ \mathbf z ''\cdot \mathbf x &{} \mathbf z ''\cdot \mathbf y &{} \mathbf z ''\cdot \mathbf z \end{array}\right] \nonumber \\ {\bar{\bar{\mathbf{J }}}}_{R2}= & {} \left[ \begin{array}{l@{\quad }l@{\quad }l} \mathbf x '\cdot \mathbf x &{} \mathbf x '\cdot \mathbf y &{} \mathbf x '\cdot \mathbf z \\ \mathbf y '\cdot \mathbf x &{} \mathbf y '\cdot \mathbf y &{} \mathbf y '\cdot \mathbf z \\ \mathbf z '\cdot \mathbf x &{} \mathbf z '\cdot \mathbf y &{} \mathbf z '\cdot \mathbf z \end{array}\right] \end{aligned}$$

A typical material parameter tensor ${\bar{\bar{M}}}={\bar{\bar{\varepsilon }}},\bar{\bar{\sigma }}, \bar{\bar{\mu }},\bar{\bar{\sigma }}_m$ is now defined with respect to the material frame $({\hat{\mathbf{x }}}'',{\hat{\mathbf{y }}}'',{\hat{\mathbf{z }}}'')$ and linked to the cells inside the dielectric. The first coordinate transformation converts the material parameters, from the material to the global frame, according to

41 $$\begin{aligned} {\bar{\bar{M}}}=\frac{{\bar{\bar{J}}}_{R1} {\bar{\bar{M}}}''{\bar{\bar{J}}}^T_{R1}}{det({\bar{\bar{J}}}_{R1})} \end{aligned}$$

The second coordinate transformation converts the parameters from the global to the local frame and this process has already been described in the preceding sections. If we consider, for example, Eqs. (7, 8), the material parameters ${\bar{\bar{\varepsilon }}},\bar{\bar{\sigma }},\bar{\bar{\mu }}, \bar{\bar{\sigma }}_m$ have to be replaced by ${\bar{\bar{\varepsilon }}}'',\bar{\bar{\sigma }}'',\bar{\bar{\mu }}'', \bar{\bar{\sigma }}''_m$. This is true for each equations in which the material parameters appear.

Our radome is characterized by the material parameters

42 $$\begin{aligned} {\bar{\bar{\varepsilon }}}''= & {} \left[ \begin{array}{l@{\quad }l@{\quad }l} 2.59 &{} 0 &{} 0 \\ 0 &{} 2.59 &{} 0 \\ 0 &{} 0 &{} 4.7 \end{array} \right] \qquad \bar{\bar{\sigma }}''= \left[ \begin{array}{l@{\quad }l@{\quad }l} 100 &{} 0 &{} 0 \\ 0 &{} 100 &{} 0 \\ 0 &{} 0 &{} 10 \end{array} \right] \nonumber \\ \bar{\bar{\mu }}''= & {} \left[ \begin{array}{l@{\quad }l@{\quad }l} 1 &{} 0 &{} 0 \\ 0 &{} 1 &{} 0 \\ 0 &{} 0 &{} 1 \end{array} \right] \qquad \bar{\bar{\sigma }}''_m= \left[ \begin{array}{l@{\quad }l@{\quad }l} 0 &{} 0 &{} 0 \\ 0 &{} 0 &{} 0 \\ 0 &{} 0 &{} 0 \end{array} \right] \end{aligned}$$

**Fig. 15:**
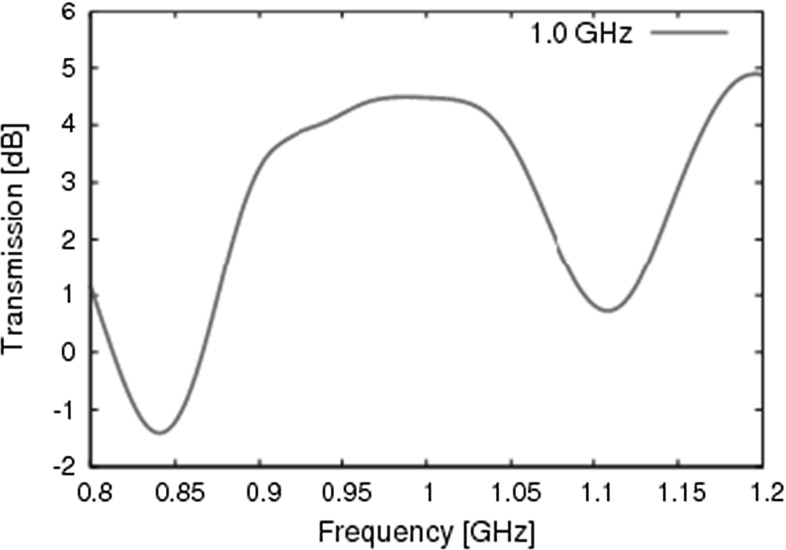
Transmission of 1 GHz pulse through a radome made out of a composite dielectric

Due to the orientation of the fibres, the properties of our composite are the same in the $\hat{\mathbf{m }}''$ and the $\hat{\mathbf{l }}''$ directions, but differ along the $\hat{\mathbf{n }}''$ axis. For our material parameters, this means that ${\bar{\bar{M}}}(1,1)''={\bar{\bar{M}}}(2,2)''\ne {\bar{\bar{M}}}(3,3)''$, and the other components are 0, for ${\bar{\bar{\varepsilon }}}'',\bar{\bar{\sigma }}''$ and $\bar{\bar{\mu }}''=\bar{\bar{I}}$ and $\bar{\bar{\sigma }}''_m=0$. Typically, a material that minimally attenuates the electromagnetic signal transmitted or received by the antenna is used. The transmission is evaluated as

43 $$\begin{aligned} T(f)=20\log _{10}\left\| \frac{\mathscr {F}\left[ E_{tot}(\mathbf r _{0},t) \right] }{\mathscr {F}\left[ E_{inc}(\mathbf r _{0},t)\right] }\right\| \end{aligned}$$

which corresponds to the ratio of the amplitude of the total electric field divided by the amplitude of the incident electric field at a point $\mathbf r _{0}$ inside the radome. The transmission of 1 GHz narrowband pulse through a composite radome is represented in Fig. 15.

## Conclusion

A Yee type algorithm has been implemented, on an appropriately generated unstructured mesh, to model electromagnetic wave scattering by bodies consisting of electrically and magnetically anisotropic and conducting dielectric materials. The implementation has been successfully validated by comparison with the results obtained using the discrete dipole approximation. The generalization from isotropic to anisotropic materials allows us now to accurately model anisotropic objects of complex geometry.

In future work, we expect to extend the method to allow for the modelling of anisotropic dispersive materials. Bi-anisotropic dispersive materials, such as metamaterials, which involve coupling electric and magnetic fields in the constitutive equations could also be included. This can be achieved using the Z-Transform [21–23]. It is also proposed to incorporate a multi-scaling procedure, to allow for the modelling of more complex materials, such as composites.
